# Assessment of the peste des petits ruminants world epizootic situation and estimate its spreading to Russia

**DOI:** 10.14202/vetworld.2018.612-619

**Published:** 2018-05-13

**Authors:** Fayssal Bouchemla, Valerey Alexandrovich Agoltsov, Olga Mikhailovna Popova, Larisa Pavlovna Padilo

**Affiliations:** 1 Department of Animal Disease, Veterinarian and Sanitarian Expertise, Faculty of Veterinary Medicine, Vavilov Saratov State Agrarian University, Saratov, Russia; 2 Department of Food Technology, Faculty of Veterinary Medicine, Vavilov Saratov State Agrarian University, Saratov, Russia; 3 Department of Epidemiology and Risk Assessment, Saratov Research Veterinary Institute - Branch of Federal Research Center for Virology and Microbiology, Saratov, Russia.

**Keywords:** forecast, incidence, outbreaks, peste des petit ruminants, risk factors

## Abstract

**Aim:**

This study focuses on the spatial dynamic associated with the spreading of the peste des petits ruminants (PPR) disease for the past decade (from the year 2007 to 2017), assesses the resulting situation in the world, and has an emphasis on Russian advantages been a PPR host.

**Materials and Methods:**

Outbreaks were confirmed and reported officially by the World Organization for Animal Health (enzyme-linked immunosorbent assay and polymerase chain reaction were used). Data contain the account number of infected, dead, and all susceptible animals in focus of infection in the period of 2007-2017. Once conventional statistical population was defined, a model was installed. Geo-information system QuickMAP was used to clear up the map disease, and through the ^@^Risk program, we got our forecasting value of future situations (by Monte Carlo method).

**Results:**

The spatial study of PPR’s occurrence and its spread was mapping according to the incidence of cases and outbreaks. Clusters demonstrated risk levels in the world in the period from 2007 to 2017 year. Based on the epizootological analysis, an assessment of PPR risk and the probability movement of infection in Russia from nearby disadvantaged countries had been carried out. A statistically significant impact of the socioeconomic system on the stationarity index was found equal to 0.63. The PPR risk of spreading could not be ignored. Nevertheless, conducting effective large-scale vaccine companies in a complex of antiepizootic activities against PPR could reduce the risk of spread of the disease up to 91.8%.

**Conclusion:**

Despite all mentioned facts above, the PPR probability can only be reduced by coordinating work of border veterinary services, as in disadvantaged as in free from this disease country, that is, what makes an effective and complete eradication of the disease could be quite realistic.

## Introduction

Peste des petits ruminants (PPR) is a highly contagious, emergent, and transboundary disease that affects goats and sheep [1,2]. The virus is transmitted by aerogenic and alimentary roads, on direct contact of healthy sheep and goats with sick animals. The first PPR report was made by Beaton, who observed the disease in Nigeria in 1930 [3]. The disease was classified as a particularly dangerous infectious disease. PPR causes colossal economic damage to goat and sheep breeding. The virus, first brought to the non-infected territory, can infect up to 100% of livestock. Mortality in the primary focus of infection can reach 100%, and in permanently infected territories - up to 50% [4,5].

A recent outbreak in India led to losses, measured in millions of dollars. A whole series of epizootics in Kenya in 2006-2008 led to the death of 1.2 million small ruminants, resulting in losses exceeding 23.5 million dollars, as well as a decrease in the production of goat milk by 2.1 million liters [2,6,7].

To study the PPR occurrence, we were obliged to determinate the condition of focus of infection within different areas because of consequences depending on various risk factors. For example, morbidity and case fatality rates vary and depending on factors such as immune status, age, species, and presence of other coinfections; they can be as high as 90-100% [8,9]. It was demonstrated by Kivaria *et al*. [9] that PPR spreading and appearance may vary from area to other depending on the region predisposition or existing factors. In fact, the question about PPR risk factors still makes a challenge that what persuades us to join this field in purpose to value happened situation in the world and have an emphasis on Russian advantages been a PPR host.

The sources of infection of the PPR virus are sick small ruminant animals that release the virus with nasal and teary outflows, as well as with feces, starting from the 1^st^ day of febrile state, without manifestation of clinical signs of the disease (2-3 days before their appearance) and throughout the period of the disease. After the disease, long-term immunity is formed in the animals, so there is no virus carrier [10].

Infection in most cases occurs with direct contact, by air and alimentary, and through other transmission factors. Goats at the age from 2 to 18 months are the most susceptible to infection. There is a predisposition to the disease of individual breeds of small cattle. Cattle are not subjected to disease, but antibody formation during contact with the virus occurs [3,5,9,11-13].

Besides goats and sheep, there are reports of clinical cases among wild small ruminants in some zoos, for example, about Laristan, Dorcas, Gemsbok, Antelopes, Gazelles, and Nubie. Antibody formation is serologically diagnosed, although against opinion still existed [8,9,14-16]. Low resistance to the virus in the external environment was noted. Goats (newborns and up to 18 months of age) are more sensitive, breed predisposition exists [13].

## Materials and Methods

### Ethical approval

As the research was epidemiological study, Ethical Committee approval was not required.

### Area of the study and samples collections

Outbreaks (serum samples were collected from clinically healthy as well as suspected animals in infected points) were confirmed and reported officially by veterinary departments, which supervise conformed geographical regions in each country in the World Organization for Animal Health (OIE). These reports showed that enzyme-linked immunosorbent assay and polymerase chain reaction were used to identify the PPR disease, taking into account number of susceptible, infected, dead animals, and PPR focus of infection from 2007 to 2017 [2].

### Statistical analysis

The model was built on the basic data. In all statistical analyses were used the level of significance set at 5% (p<0.05). The ^@^Risk program helped us to build our forecasting by Monte Carlo method (triangular distribution). Moreover, through the Geo-information system, QuickMAP has processed clustering analysis of cases and outbreaks incidence (amount of cases/or outbreaks by one focus of PPR infection) with mapping.

The predisposing factor for the emergence and spread of PPR is socioeconomic conditions. To analyze the structure of the PPR range, we used hypothesis testing methods, the *χ*^2^ criterion for multivalued populations, which allows us to identify significant prerequisites for the disease and impact information indicator (III) of the degree of socioeconomic conditions influence factor on the level of tension of the epizootic situation by the formula [17,18]:


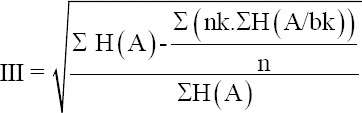


Where: (n_k_ H (A/b_k_))/n - the entropy of accidental diversity

ΣH (A) - general entropy.

### Tests

Once conventional statistical population was defined (an observational study), a model was built. Moreover, by this way, we could know not only the relative risk (RR) of the incidence, but also we could have assessment and measurement of risk factors. To calculate the connection degree of the dynamic of PPR stationary, geographical and socioeconomic conditions were used methods of hypothesis testing, criterion *χ*^2^, and calculation of the influence force of various systems: The entropy of random diversity and total entropy of the complex (Table-1).

**Table-1:** Influence of socioeconomic conditions on the tension of the PPR situation (stationary level).

Zones	Stationary value				ΣF	P_1_	ΣE	ΣE*Σf

	[0.1-0.2]	[0.2-0.3]	[0.3-0.4]	[0.4-0.5]				
Eastern Europe (Georgia)	1				1	0.052	0	0
	1							
	0							
Central Asia (Mongolia and Tajikistan)	1	1			2	0.105	1	2
	0.5	0.5						
	0.5	0.5						
East Asia (China, Bhutan, Moldova)	1	1		1	3	0.157	1.583	4.749
	0.33	0.33		0.34				
	0.527	0.527		0.529				
Mediterranean (Algeria, Israel, Egypt, Tunisia, Morocco)		2	1	2	5	0.263	1.52	7.6
		0.4	0.2	0.4				
		0.528	0.464	0.528				
Central Africa (Zambia, Uganda, Tanzania, Liberia, Kenya, Comoros, Angola, Gabon)	7	1			8	0.421	0.543	4.344
	0.875	0.125						
	0.168	0.375						
ΣF	10	5	1	3	19			
P_2_	0.526	0.263	0.052	0.157				
H (A)	0.487	0.506	0.222	0.419				

f=Frequencies, number of objects in each class, 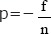 frequency fractions, E=H=−p log_2_ P - particular entropies by class, found for each fraction (with error probability=0.05), PPR=Peste des petits ruminants

## Results and Discussion

Focus epizootic of PPR infection is manifested in isolated outbreaks in the affected areas. The spread of the disease is mainly due to the movement of livestock and the small ruminant’s trade. The PPR incidence is lower in dry climate with high air temperature and, accordingly, the highest incidence is observed in the areas that are characterized by moderate temperature conditions with high air humidity.

In 2017 and according to the OIE data, only 16 countries have officially announced about natural outbreaks, but just the Maldives and Tajikistan had defined own situation, although the virus was found in more extensive areas. The geographic location of these regions shows that cases of death of sheep and goats are associated with the spread of the PPR from China and, of course, African continent [2,10,12,19].

**Figure-1:**
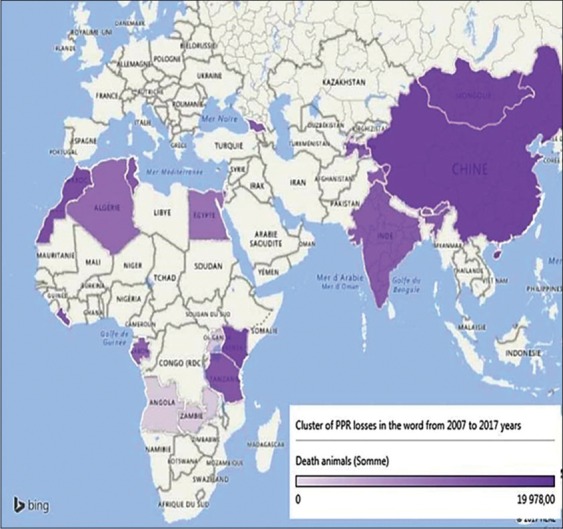
Clustering of livestock mortality caused by peste des petits ruminants in the world in 2007-2017 [Source: Figure was made with the help of @Risk program].

In the period from 2007 to 2017, PPR had involved, many countries (16 countries) had reported the detection of this virus in their territories in 2017 (including 10 Asian countries), and 56 countries had made such reports in 2016 (15 African), with varying enzootic frequency (0.1-0.4). PPR registered in 649 points with 150 heads as an average focal nature of infection. The prevalence of long-standing infection exceeded 28%. Morocco and China suffered from most of all 80% of cases, with an average lethality of 43.7% (1.53-95.63). Clustering of the fallen animal’s data showed that about 50% of the economic damage fell in China, and less than in Mongolia (26% of the animals died) (Figure-1).

**Figure-2:**
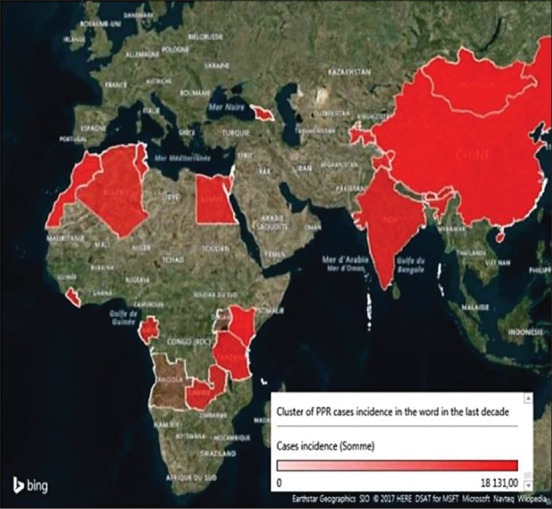
Cluster of the incidence of the peste des petits ruminants cases in the world in the past 10 years [Source: Figure was made with the help of @Risk program].

**Figure-3:**
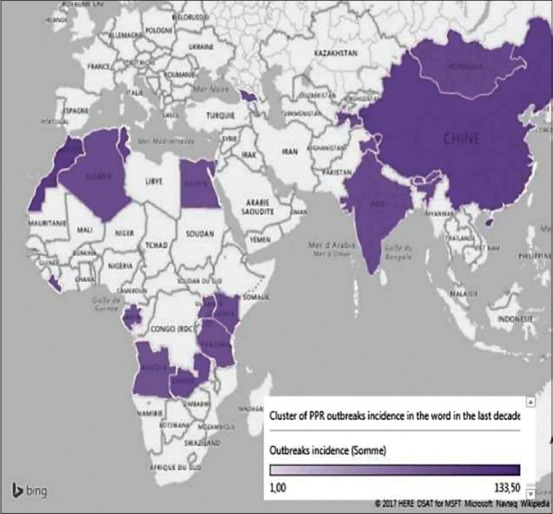
The incidence level of the peste des petits ruminants outbreaks in the past 10 years [Source: Figure was made with the help of @Risk program].

**Figure-4:**
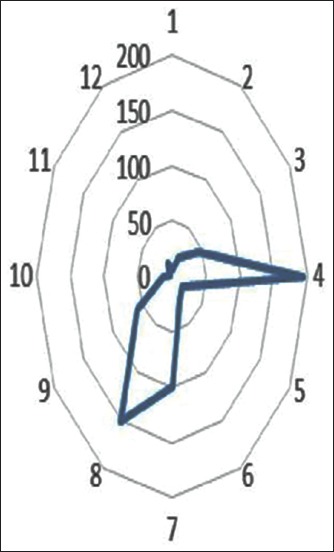
The monthly number of peste des petits ruminants outbreaks in the world (2007-2017).

The disease emergence rate was assessed by calculating its incidence of case and outbreaks. Cartographic analysis of its cluster (Figures-2 and 3) shows that PPR virus circulation seems to a triangular field: China, Algeria, and Zambia (including their adjacent territories), which indicates that when the disease emerged, the probability of regional coverage could have estimated precisely. While other parameters, influencing on the intensity of the epizootic process are summarized in Table-1 and Figure-4.

PPR is characterized by its seasonal prevalence. The distribution of unfavorable points according to the months of the year has two leaps. The first peak falls on the 4^th^ month (April) and the second (its size is greater than the first one) for the 8^th^ month (August) (Figure-4). This time coincided with the pasture period (direct contact increases) and the maximum activity of the virus carriers-vectors. Their presence and activity strengthen the course of the epizootic process.

Analysis of the structure of the PPR area had provided the degree assessment of the influence of socioeconomic background factor on the incidence and nature of the spread of the disease, as well as on the tension of the epizootic situation. Statistical result taken in the proposed zoning (according to the published data by Food and Agriculture Organization [FAO]) over the period from 2007 to 2017 is shown in Table-1.

The informational indicator of influence can take values from 0 to 1. Socioeconomic conditions, along with natural features, have a significant influence on the formation of the geography of the epizootic situation in the world. Their study has the most important for revealing the peculiarities of the epizootic situation in different regions since they tend to change constantly. Qualitative indicators directly depend on the analysis of factors that include socioeconomic background, zoning of territories according to the level of epizootic risk, and probable consequences of epizootics [6,17,18].

From Table-1, the impact information indicator (III) was found:

n = F=19

(n_k_*H (A/b_k_)) = Σ (ΣF*ΣE)=18,693

E_y_ = ΣH (A)=1,634, E_z_=0,998

E_x_ = Э_y_-Э_z_=0,636

III = 0,631.

The calculations established statistically significant system influence of social and economic parameters on the index of stationarity, which is about 0.63 (direct and strong connection).

The obtained data convincingly confirm a larger role of social and economic conditions in maintenance and development of PPR in the whole world. An epizooty in East Asia, in particular, China, was the main feature of this disease evolution. In this region, the indicator of the general entropy complex is about 1.58, and there is a high rate in the Mediterranean area 1.52, too. It had noticed that only two subjects have stationarity 0.4 and more.

Using similar approach, we had defined that III of geographical and climatic conditions is up to 0.62. In recent research, conducted in China, was shown that climate conditions exert a significant impact on the PPR emergence [19,20].

PPR virus reaches animal blood (leukocytes and erythrocytes) even 12 h before the beginning of fever and throughout the entire period of illness with a various concentration 10^2^-10^4^ TCD_50_/ml [2]. Therefore, blood-sucking carriers (vectors) can only reinforce epizootic process during a season of their active presence.

In Mongolia, in 2016, about 36 thousand animals (70% at goats, 20% at sheep, and 10% wild animals) were got by PPR, at the same time, there were reports about deaths of 3999 of wild animals, and it was 100% of mortality. All these cases confirm an intensive circulation of a virus among livestock, and in general, the expected risk of the cross-border migration of infected wild animals. Although there is a challenge opinion about their participation in the process, risk remains, and it was presented by Dhar *et al*. [2,8,9,13].

RR of PPR in goats by comparison with sheep was over one and reached 3.66 that ought to be considered a high-level risk of infection diffusion among this livestock. Moreover, the excessive risk of PPR emergence in goat farming is about 0.59. This point was developed in similar research, carried out by the Al-Majalia *et al*. [11], in a special geographic region.

Based on PPR data for 2007-2017 in the world and the epizootological analysis, the forecast of the development of epizootic process of PPR was made. Epizootological forecasting was carried out by the Monte Carlo method using the software - ^@^Risk Professional Edition.

The probabilistic method of sampling is the basis of the Monte Carlo method that imitates the effect of accident, and then, uses these data for selection of the general data array offered by the model. Calculation of the PPR prognostic values was performed with the use of triangular distribution (in 1000 iterations).

The probable average annual quantity of the general cases of illness and death of animals in farms was used for risk assessment. Calculations were carried out, according to the argument that the high expected number of the infected and/or fallen animals means high expected risk.

**Figure-5:**
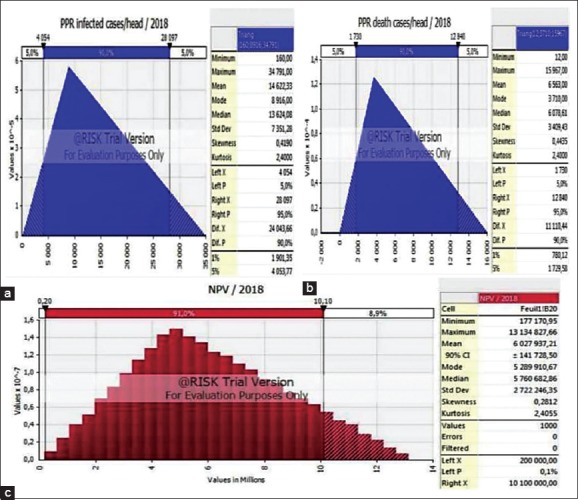
Monte Carlo model and peste des petits ruminants forecast for 2018. (a) Total annual risk peste des petits ruminants (PPR) assessment in the world (number of cases) for 2018. (b) Total forecasted average of animal dying from PPR in the world for 2018. (c) Estimation of sensitivity analysis of our mode-net present value - net present value [Source: Figure was made with the help of @Risk program].

Figure-5a demonstrates that the average annual case number for 2018 will be 14,622 cases by the Monte Carlo method (when using triangular distribution). It was similarly defined that lethality reaches 44.88%, which corresponds to 6563 heads of forecasted average quantity of the expected fallen goats and sheep for 2018 in the world (Figure-5b). In addition, it was calculated that the total number of animals under the PPR risk for 2018 would be more than 6 million heads. This indicator corresponds to a prevalence not more than 0.5%.

To assess the accuracy of our analysis, the net present value (NPV) of model (NPV-indicates healthy animals) (Figure-5c) was calculated, using recorded data of the cases number and animals fallen from PPR in the world over the past 10 years. The histogram (Figure-5c) shows that there is a small chance (0.1%) that NPV approaching zero. In contrast, the NPV at the point of 10.1 million is an accurate indicator with a probability of 91.8%. A positive indicator was formed from conducting effective large-scale vaccine companies and complex of antiepizootic actions against PPR, especially the expected of them in the future. Positive results are possible by the cooperative work of FAO, OIE, and other organizations. However, the question of the disease spread remains important, as while working on the interactive graphic, the probability became 0.6% and 39.3% for the minimum and mode NPV value. At the NPV calculating, the number of all animals includes them, who have been recuperated after sickness, i.e., a part of these values indicates expected animals to be fallen out of PPR.

**Figure-6:**
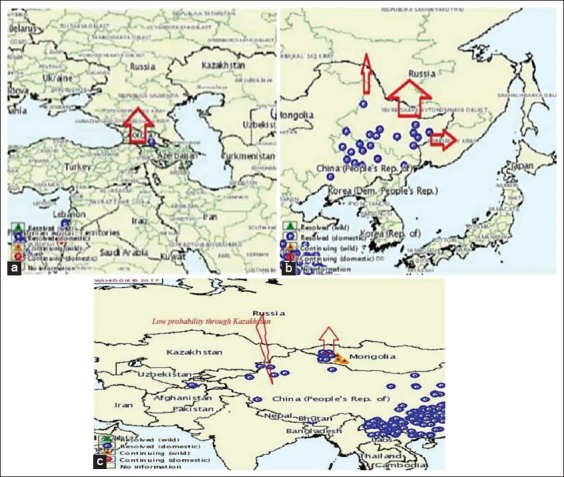
Probability peste des petits ruminants risk occurring to Russia. (a) Probability of the peste des petits ruminants (PPR) risk occurring from Georgia (Republic of North Ossetia-Alania). (b) Russian regions with a significant probability of PPR occurrence through China (Primorsky Krai, Amur and Jewish Autonomous region). (c) The PPR situation at Mongolian border and the risk of its occurrence on the territory of the Russian Federation in 2018.

Taking into account, the geographical location of the Russian territory, which borders with the endemic territory, expected risk of disease occurrence is very high. Visual analysis of geographical maps shows that the main entrance gate of PPR virus to Russia is Mongolia, China, and Georgia. The directions of the spread of infection while animals and their products come cross borders are shown in Figure-6a-c.

An important PPR reservoir (with low to moderate level of risk) is the number of susceptible wild animals that are uncontrolled and asymptomatic virus carriers. The important risk to see the infection in such Russian regions as Altai, Tyva, and Buryatia is through Mongolian epizootic gate (Figure-6c).

**Figure-7:**
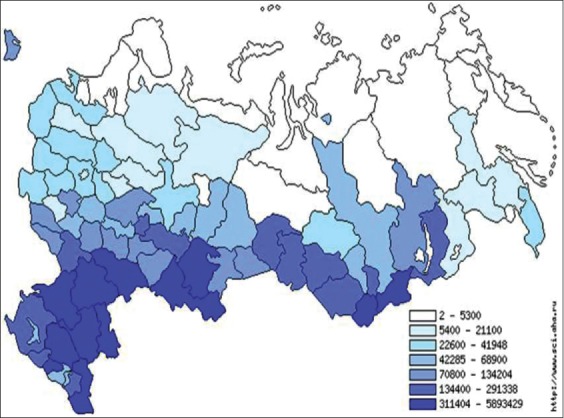
Intensity of sheep and goats distribution in the territory of the Russian Federation (2017).

As shown in Figure-7, the main intensity of susceptible animal’s distribution in the territory of Russia is in the southern part of the country that increases the risk of spread of PPR from China, Mongolia, Georgia, and Tajikistan.

The intensive circulation of the virus among the wild stock provides in general, high expected risk of PPR, in the territory of Russia from the uncontrolled transboundary migration of these animals.

## Conclusion

The probability of PPR virus spreading to the southern regions of Russia is quite high; insofar as the closed border with infected countries, having a high degree of the PPR episodic process. According to the forecasting, unfortunately, PPR will be expected in the next year. Despite all facts were mentioned above, the PPR probability could be effectively reduced by coordinating the work of border veterinary services, as disadvantaged as free from this disease country, that is, what makes an effective and complete eradication of the disease could be quite realistic.

## Author’s Contributions

FB designed the work. All authors conducted the research work. OMP and LPP done data analysis and manuscript drafting under the guidance of FB and VAA. All authors read and approved the final manuscript.
